# Characterization of the first symptoms of multiple sclerosis in a Brazilian center: cross-sectional study: Caracterização dos primeiros sintomas de esclerose múltipla em um centro brasileiro: estudo transversal

**DOI:** 10.1590/1516-3180.2016.0200270117

**Published:** 2017-04-03

**Authors:** Vitor Breseghello Cavenaghi, Fernanda Martinho Dobrianskyj, Guilherme Sciascia do Olival, Rafael Paternò Castello Dias Carneiro, Charles Peter Tilbery

**Affiliations:** I Undergraduate Student, Faculdade de Ciências Médicas da Santa Casa de São Paulo (FCMSCSP), São Paulo (SP), Brazil.; II MD. Neurologist, Department of Neurology, Faculdade de Ciências Médicas da Santa Casa de São Paulo (FCMSCSP), São Paulo (SP), Brazil.; III MD, PhD. Neurologist, Department of Neurology, Faculdade de Ciências Médicas da Santa Casa de São Paulo (FCMSCSP), São Paulo (SP), Brazil.

**Keywords:** Multiple sclerosis, Academic medical centers, Symptom assessment, Illness behavior, Cranial nerves

## Abstract

**CONTEXT AND OBJECTIVE:**

Multiple sclerosis (MS) is a chronic, immune-mediated and degenerative central nervous system (CNS) disease with well-established diagnostic criteria. Treatment can modify the course of the disease. The objective of this study was to describe the initial symptoms of multiple sclerosis in a Brazilian medical center.

**DESIGN AND SETTING:**

Descriptive study, conducted in a Brazilian reference center for multiple sclerosis treatment.

**METHODS:**

Data on 299 patients with confirmed diagnoses of MS were included in the study. Their medical files were evaluated and the data were analyzed.

**RESULTS:**

The most common symptom involved the cranial nerves (50.83%) and unifocal manifestation was presented by the majority of this population (73.91%). The mean time between the first symptom and the diagnosis was 2.84 years. Unifocal symptoms correlated with longer time taken to establish the diagnosis, with an average of 3.20 years, while for multifocal symptoms the average time taken for the diagnosis was 1.85 years. Unifocal onset was related to greater diagnostic difficulty.

**CONCLUSIONS:**

MS is a heterogeneous disease and its initial clinical manifestation is very variable.

## RESUMO

**CONTEXTO E OBJETIVO:**

A esclerose múltipla (EM) é uma doença crônica do sistema nervoso central (SNC) imunomediada e degenerativa, com critérios diagnósticos bem estabelecidos. O tratamento pode modificar o curso da doença. O objetivo deste estudo foi descrever os sintomas iniciais da esclerose múltipla em um centro médico brasileiro.

**TIPO DE ESTUDO E LOCAL:**

Estudo descritivo, conduzido em um centro médico de referência no tratamento de EM no Brasil.

**MÉTODOS:**

Foram incluídos no estudo dados de 299 pacientes com diagnóstico confirmado de EM. Seus prontuários foram avaliados e os dados foram analisados.

**RESULTADOS:**

O sintoma mais comum encontrado envolveu nervos cranianos (50,83%) e a manifestação unifocal foi apresentada pela maioria da população estudada (73,91%). O tempo médio entre o primeiro sintoma e o diagnóstico foi de 2,84 anos. O sintoma unifocal foi relacionado com maior tempo para o estabelecimento do diagnóstico, com uma média de 3,20 anos; enquanto para os sintomas multifocais, a média foi de 1,85 anos para o diagnóstico. O início unifocal foi relacionado a maior dificuldade de diagnóstico.

**CONCLUSÕES:**

EM é uma doença heterogênea e sua manifestação clínica inicial é muito variável.

## INTRODUCTION

Multiple sclerosis (MS) is a chronic, immune-mediated and degenerative central nervous system (CNS) disease that leads to injury to myelin and axons and results in different neurological signs and symptoms, with dissemination over space and time.^1^

There are no biological markers for MS. Today, the diagnosis is made from clinical findings, lesions identified on magnetic resonance imaging (MRI), presence of oligoclonal bands and/or high levels of immunoglobulin G (IgG) in the cerebrospinal fluid (CSF), as described in the revised McDonald criteria in 2010.^2^ The most commonly presented form of MS is relapsing and remitting. In this, neurological symptoms or lesions are followed by periods of clinical improvement or latency. On the other hand, the progressive form can present either at the beginning of the disease (primary progressive), or after years of the relapsing-remitting form (secondary progressive).^3^

Despite these known diagnostic criteria, there is difficulty in establishing the diagnosis of MS and its onset is usually neglected since these initial symptoms may resolve spontaneously. This situation leads to delayed diagnosis, which consequently delays the treatment and has a negative impact regarding the speed of progression of the disease, and its prognosis.^4^ Thus, knowledge of the initial manifestations of MS has great epidemiological value, since it can contribute towards decreasing the time between MS onset and treatment, and may slow the progression of the disease.

## OBJECTIVE

The aim of this study was to identify the initial symptoms of MS in a group of patients and to identify the symptoms that are related to probable diagnostic difficulty (characterized by longer time between symptoms and diagnosis).

## METHODS

This study was previously approved by our institution’s Ethics Committee, under protocol no. 075/12. The patients selected for the study were outpatients at Centro de Atendimento e Tratamento da Esclerose Múltipla (Catem), São Paulo, Brazil. 

This was a cross-sectional study, in which patients at Catem were selected, in accordance with the inclusion and exclusion criteria specified below. 

Data were collected from information contained in medical records and from magnetic resonance imaging (MRI) accessed using IMPAX (Agfa HealthCare NV, Belgium). They were analyzed descriptively using simple statistical ratios consisting of means, standard deviations and percentages. 

The symptoms were grouped as follows: motor, sensory, cranial nerve, prodromal, urinary system and balance. The patients were divided between unifocal presentation, when they had one first symptom, and multifocal presentation, when they presented two or more symptoms upon enrollment.

### Inclusion criteria:

- Diagnosis of MS (made between 1984 and the present day, fulfilling the diagnostic criteria that were current at the time of diagnosis);
- Presence of information in the medical records regarding: date of symptom onset, symptoms lasting for more than 24 hours and date of diagnosis;
- Initial manifestations dissociated from any other medical condition.

### Exclusion criteria:

- Diagnoses of demyelinating diseases other than MS;
- Not meeting the criteria for MS.

## RESULTS

Among the 563 patients, 299 met the inclusion criteria and were enrolled in this study. If the patients met the inclusion criteria, they were included as they were attended, sequentially. The population consisted of 214 women (71.57%) and 85 men (28.42%). The average age at the onset of symptoms was 26.99 years (standard deviation, SD ± 9.68), with a minimum of 7 and maximum of 58 years. The median time to diagnosis was 2.74 years and the maximum was 26 years. The minimum was a diagnosis at the time of the initial symptoms. The minimum age at diagnosis was 7 years and the maximum was 63 years.

Of these patients, 221 (73.91%) had unifocal symptoms, 77 (25.75%) had multifocal symptoms and one (0.33%) had no reported symptoms. These 299 patients reported 392 symptoms: 79 (26.42%) had motor symptoms; 96 (32.10%) had sensory symptoms; 152 (50.83%) had symptoms involving the cranial nerves; 23 (7.69%) had prodromal symptoms (like headache, fatigue, asthenia, nausea/vomiting, malaise, low back pain and depression); 40 (13.38%) had symptoms involving their balance; and 2 (0.67%) had urinary system symptoms. The symptoms and their descriptions are presented in Table 1.

**Table 1:**
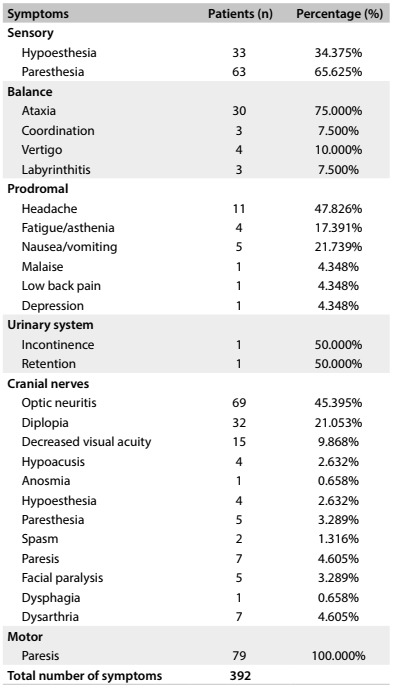
Symptoms presented

The mean time between the first symptom and the diagnosis was 2.84 years. Unifocal onset was correlated with longer time to establish the diagnosis, with an average of 3.20 years (minimum of simultaneous diagnosis and maximum of 26 years after the initial symptom). For the patients with multifocal symptoms, the diagnosis was reached on average 1.85 years after symptom onset (minimum time coincident with the clinical manifestation and maximum time to diagnosis of 12 years).

## DISCUSSION

Ashtari et al.^5^ evaluated 123 MS patients with a mean age of 27.7 years (SD = 8.06) with onset after they reached 16 years of age. Among them, 29.3% initially had optic neuritis, 36.6% paresthesia, 18.7% cerebellar or brainstem symptoms, 14.6% motor symptoms and 0.8% other symptoms. Barkhof et al.^6^ found, among 74 MS patients, that 54% had optic neuritis at the onset, 16% cerebellum or brainstem symptoms and 30% spinal symptoms.

In another study, 97 patients with clinically isolated syndrome (CIS) were followed for two years, and 59 were found to fulfill the revised McDonald criteria for MS after 10.1 ± 4.2 months; 37 (38.1%) fulfilled the criteria through radiological parameters and 21.7% from a second clinical event. The initial manifestations of the patients who fulfilled the criteria for MS were related to the optic nerve in 11 (16.18%), cerebellum and brainstem in 15 (22.06%), spinal cord in 19 (27.94%) and supratentorial region in 18 (26.47%), and 5 (7.35%) of them showed multifocal manifestations.^7^

In a study conducted in Denmark, 7,548 patients with diagnoses of MS that were established between 1949 and 1990 were assessed regarding optic neuritis as the initial manifestation of the disease. Among the 6,923 patients whose initial manifestations were known, optic neuritis marked the onset of MS in 1,282 cases (19%). Among the patients for whom optic neuritis was not present at the onset of MS, the mean ages at the time of manifestation and diagnosis were 6.1 years and 4.2 years.^8^

In the present study, the initial manifestations were divided into more categories (motor, sensory, cranial nerve, prodrome, balance and urinary system manifestations), to better describe what was observed clinically. Symptoms like anosmia or dysphagia were presented by small numbers of patients and these manifestations were dissociated from any other medical condition. Therefore, they were also considered to be onset symptoms of MS, thus illustrating the heterogeneity of MS.

The average time to diagnosis was 2.84 years shorter than the time described by Sorensen et al. However, this may be explained by the evolution of MS diagnostic criteria over recent years, through use of neuroimaging and oligoclonal bands, for example.^9^ In our study, we used the diagnostic criteria that were current from 1984 to 2015 and we believe that if the inclusion criteria had included the revised McDonald diagnostic criteria, the results would probably have been different.

The importance of early diagnosis lies in early establishment of use of immunomodulatory drugs, given that their use correlates with a decreased rate of progression of the disease.^4^ The PRISMS-4 study,^9^ for example, demonstrated the benefits of early treatment of MS. Patients diagnosed with MS were divided into two groups: the first received placebo, followed by two years of treatment with 22 mcg or 44 mcg of interferon-β 1a, while in the second group, the patients received treatment for four years in a row. After four years, the first group showed greater development of the disease, as assessed by Expanded Disability Status Scale (EDSS).

## CONCLUSION

MS is a heterogeneous disease and its clinical manifestations are quite variable. The average time between the onset of MS symptoms and the diagnosis was 2.84 years, and this time was longer among patients who presented unifocal symptoms (3.20 years) than among those who presented multifocal symptoms (1.85 years). Unifocal onset was correlated with greater diagnostic difficulty. The most common initial symptoms related to cranial nerves (38.77%), followed by sensory symptoms (24.49%) and motor symptoms (20.15%). MS is a heterogeneous disease and its clinical manifestation is quite variable.
